# Laparoscopic Pectopexy—CUSUM Learning Curve and Perioperative Complications Analysis

**DOI:** 10.3390/jcm10051052

**Published:** 2021-03-04

**Authors:** Paulina Szymczak, Magdalena Emilia Grzybowska, Sambor Sawicki, Dariusz Grzegorz Wydra

**Affiliations:** Department of Gynecology, Gynecological Oncology and Gynecological Endocrinology Medical University of Gdańsk, Smoluchowskiego 17, 80-214 Gdańsk, Poland; paulina.szymczak@gumed.edu.pl (P.S.); sambor.sawicki@gumed.edu.pl (S.S.); dwydra@icloud.com (D.G.W.)

**Keywords:** apical prolapse, Clavien-Dindo, cumulative sum, pectopexy, laparoscopy, pelvic organ prolapse

## Abstract

The study aimed to examine the learning curve and perioperative complications for laparoscopic pectopexy (LP). A total of 60 women with stage II–IV apical prolapse who underwent LP were dichotomized into groups: LSH(+) with concomitant laparoscopic supracervical hysterectomy (LSH), LSH(−) after previous supracervical/total hysterectomy. Operative time, estimated blood loss and hospitalization length were evaluated with cumulative sum (CUSUM) analysis and the Kwiatkowski–Phillips–Schmidt–Shin (KPSS) test, separately for two surgeons (A and B). Intraoperative and perioperative complications according to the Clavien–Dindo (C–D) classification were analyzed. Mean operative time, change in hemoglobin level, and postoperative hospital stay were 143.5 ± 23.1 min—1.5 ± 0.5g/dL and 2.5 ± 0.9 days, respectively. LSH during pectopexy was associated with longer operative time (*p* = 0.01) but not with higher intraoperative bleeding or prolonged hospital stay. Severe complications rate was low (1.7%) with one bowel injury in LSH(−) (C–D grade IIIb). No C–D grade II, IV and V complications were found. Conversion to open pectopexy, return to the operating room or blood transfusion were not required. The KPSS test showed that a steady operative time for Surgeon A was achieved after 28 procedures. A proficiency for laparoscopic pectopexy based on CUSUM analysis was observed after 38–40 procedures.

## 1. Introduction

Pelvic organ prolapse (POP) is a common health problem, with the prevalence up to 50% when based upon vaginal examination. The health-related quality of life among women with POP is deteriorated as compared to the age-standardized population. After surgery 90% patients perceived their condition to be improved during the 2-year follow-up [1]. Apical vaginal support is considered the keystone of pelvic organ support [2], although it is the least frequent of all POP types with the range of 5–15%. It can be corrected by abdominal, vaginal and minimally invasive surgery [2,3]. Rapid advances in reconstructive pelvic floor surgery, including robotic techniques, have been a source of interest for numerous researchers [4,5,6,7,8,9]. The aims of the surgery include restoration of normal vaginal anatomy, bladder and bowel function, and restoration or maintenance of sexual function [2]. A sacrocolpopexy (LS) is a confirmed and effective standard for post-hysterectomy vault prolapse [4], and sacrocervicopexy is believed to additionally preserve adequate vaginal length and reduce the risk of erosion [9]. Objective success rates of LS and sacrocervicopexy of up to 92% have been reported, and mean patient satisfaction level was as high as 94.4% [10]. With increasing surgical experience, conversion rates and operative time for LS have decreased over the years [10]. However, limitations such as patient obesity [11] or obstructed defecation syndrome caused by injury of the hypogastric nerves and reduced pelvic space [10] have brought new techniques to light, e.g., laparoscopic pectopexy (LP). First reported in 2010, LP has since become the focus of clinical research [11,12,13,14,15,16,17,18]. In a randomized single-center trial comparing LP with LS, comparable if not equally effective POP treatment, with no severe complications, was demonstrated [14]. In studies on surgical learning curves, the cumulative sum (CUSUM) analysis and the Kwiatkowski–Phillips–Schmidt–Shin (KPSS) test are usually applied [6,7,8,19]. CUSUM is a type of control chart used to monitor small shifts in the process mean, it has been adopted for the medicine to express graphically the surgeon performance [19,20].

The literature offers a modest number of reports about the learning curve for POP surgery, and those are mainly limited to sacrocolpopexy [5,6,7,8,21,22,23]. At present, more studies on LP are published, but none of them focused on the learning curve or specified the number of procedures needed to achieve proficiency [13]. Apart from the initial German group [11,12,13], two centers in Turkey [15,16] and one in Romania [17], and South Korea [18] conduct the procedure and publish their findings. Analysis of learning curves allows for more accurate evaluation of surgical training or implementation of new procedures [7].

Complications which may occur constitute an important aspect of surgery-related safety [10]. The Clavien–Dindo (C–D) classification, is a reliable way of reporting perioperative adverse events [24]. It has been adopted in gynecological procedures, including vaginal native tissue repair for POP, laparoscopic and robotic-assisted interventions. It is distinguished from other classifications by including therapy needed to correct the complications. However, it has not been widely used in studies on LP [25].

The objective of the study was to use the KPSS test and CUSUM analysis to investigate the LP learning curve; to assess LP in daily clinical practice with regard to adverse events occurring during surgery and in the early postoperative period with C–D classification.

## 2. Materials and Methods

An observational study was conducted in 60 consecutive symptomatic women with apical prolapse II–IV Pelvic Organ Prolapse Quantification (POP-Q) stages, who underwent LP in the university-based medical center [26]. Medical history was taken, urogynecological examinations were performed in accordance with the standards of the International Continence Society. Patient characteristics such as age, menopausal status, parity, POP-Q stage, previous POP surgeries and body mass index (BMI), as well as surgical information (date, duration of the surgery, concurrent procedures), pre- and postoperative hemoglobin level, length of hospital stay, perioperative complications were collected and analyzed. Estimated blood loss was calculated by measuring the difference between pre- and postoperative hemoglobin levels. Operative time was estimated as time from the first incision until the last suture. Length of hospital stay was defined as the interval between surgery and discharge from the hospital. According to our center standard protocol, the minimal stay after this procedure is 2 days. Perioperative complications were specified using five grades of severity, in accordance with the general rules of the C–D classification [25]. The data on all operative reports, discharge summaries, outpatient notes, and any emergency room visits were reviewed to assess the complication rates within 30 days postoperatively. Data were presented separately for surgeons A and B. Before introducing LP into our center, surgeons A and B had 25 and 13 years of laparoscopic experience, respectively, including laparoscopic oncological procedures. Additionally, the patients were dichotomized into two groups: LSH(+)—women with concomitant laparoscopic supracervical hysterectomy (LSH) and LSH(−)—women after previous supracervical or total hysterectomy. The study investigated the perioperative complications after LP in patients with history of total or supracervical hysterectomy as compared to concomitant LSH.

### 2.1. Operative Procedure

**Figure 1:**
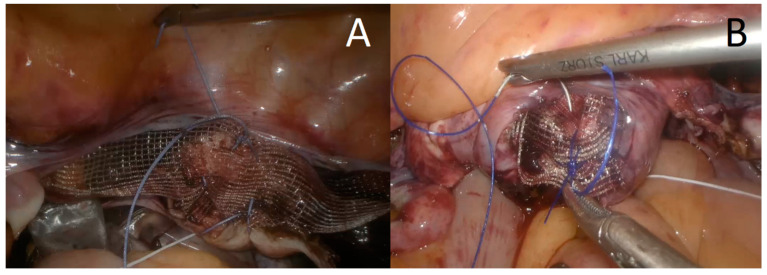
Mesh attachment during laparoscopic pectopexy to the cervix (**A**) or vaginal cuff (**B**).

LP starts with creating the pneumoperitoneum with the Veress needle, inserting the trocars, video telescope and laparoscopic instruments. If the uterus was present a supracervical hysterectomy was conducted, accompanied by opportunistic uni- or bilateral salpingo-oophorectomy or bilateral salpingectomy to prevent ovarian cancer [27]. The organs were removed from the abdominal cavity in endo-bags. In-bag removal or morcellation of the uterine corpus were performed to reduce the risk of dissemination of an unrecognized neoplastic process. Polypropylene mesh sized 20/35 × 159 mm was attached symmetrically to the iliopectineal ligaments (with non-absorbable, braided sutures) and fixed to the vaginal cuff or cervix (with 0 polypropylene monofilament or braided polyester non-absorbable sutures, respectively) (Figure 1). The procedure was performed as described previously by Noé et al. [13,28]. The mesh was covered with peritoneum using continuous absorbable suture in an endoscopic suturing technique. In two patients concomitant perineal repair was performed.

### 2.2. Statistical Analysis

Continuous variables were expressed as mean and standard deviations, categorical variables as percentages of the total group. In order to test for significant differences in group characteristics, a series of Mann–Whitney U tests was performed for continuous variables, or Pearson’s chi-square test for ordinal or nominal variables. The *p*-value of < 0.05 was considered as statistically significant, all tests were two-sided.

CUSUM analysis of learning curves was organized in two steps approach. 

Step 1. Regression models for operative time, estimated blood loss and length of hospital stay.

Before establishing regression models, an exploratory analysis was conducted. For each surgeon, a set of independent variables was chosen based on its significant relation to the dependent variables (operative time, estimated blood loss, and length of hospital stay). A series of linear regressions were conducted to explain variability of the dependent variables. Regression models were necessary due to the independent variables [BMI, sum of concurrent procedures, sum of previous POP surgeries, and the LSH(+) or LSH(−) group membership], which influenced the dependent variables described above.

Step 2. CUSUM charts depicting learning curves for both surgeons.

Predicted values for operative time, estimated blood loss, and length of hospital stay were used in step 2 as input data in CUSUM charts building. The CUSUM control chart is the sum of deviations and verifies any mean deviation in the continuous process or time series. A two-sided CUSUM scheme, which detected a shift in either direction from the target mean, was created. The mean operative time for both surgeons was used as a reference value. The centerline on the CUSUM chart is zero (i.e., “on target”). When a surgery took more or less time than the mean operative time, the graph rises or falls with the absolute difference, respectively. This provides information that the process has moved significantly off the target and needs to be re-adjusted to the target value. Success is achieved when the variability of the operative time results decreases or is kept close to the average surgeon’s operative time.

CUSUM charts were calculated in R software, using the quality control charting package and statistical process control. To test when learning curve indicates a learning phenomenon, the KPSS test was conducted. The KPSS test verifies the hypothesis that the investigated time process has a stationary trend [7,19].

The Local Ethics Committee approved the study protocol (No. NKBBN/192/2019, 10 April 2019). Declaration of Helsinki was followed. All patients provided written informed consent.

## 3. Results

### 3.1. Characteristics of the Study Population and Surgical Procedures

Out of the 60 patients with apical prolapse, 1 (1.7%) was diagnosed with POP-Q II stage, 44 (73.3%) with POP-Q III, and 15 (25.0%) with POP-Q IV. Mean age and BMI were 62.6 ± 8.5 years and 27.6 ± 4.1 kg/m^2^, respectively. Mean operative time and hospital stay after surgery were 143.5 ± 23.1 min and 2.5 ± 0.9 days, respectively. Mean pre- and postoperative hemoglobin levels were 13.5 ± 0.9 g/dL and 12 ± 0.9 g/dL, respectively (*p* < 0.001). The study group included 19 and 41 patients in the LSH(−) and LSH(+) groups, respectively. In the LSH(−) group 8 (42.1%) patients have had supracervical hysterectomy, 4 (21.1%) total vaginal hysterectomy and 7 (36.8%) total abdominal hysterectomy in their past (Table 1). 

LSH during pectopexy was associated with higher operative time (*p* = 0.01) but did not prolong hospital stay (*p* = 0.81) and was not associated with increased intraoperative bleeding (*p* = 0.74). LSH(−) had significantly higher preoperative POP-Q stage as compared to LSH(+) (*p* < 0.001). Surgeon A performed 44 (73.3%) and surgeon B 16 (26.7%) surgeries. Patients operated on by two surgeons did not differ significantly in terms of preoperative POP-Q stage, age, and BMI, as well as estimated blood loss, and length of hospital stay (*p* > 0.05). Concurrent procedures were more frequently performed in LSH(+) group (*p* < 0.0001) with bilateral salpingo-oophorectomy as the most common procedure (*p* = 0.001). The uterus, if small, was removed in a bag through one of the trocar incisions in the suprapubic area in 32 (78%) patients or using in-bag uterus morcellation in 9 (22%) patients. In-bag morcellation did not prolong operative time as compared to in-bag removal (*p* = 0.62). Concurrent procedures and perioperative complications are presented in Table 2.

### 3.2. Learning Curve Analysis

The regression analysis showed that for surgeon A, the operative time was prolonged by BMI and sum of the concurrent procedures, while for surgeon B, the operative time was shortened by LSH(−) group membership. Only for surgeon B was the change in hemoglobin level increased by BMI. The hospital stay was unrelated to the variables in the regression model. The sum of previous POP surgeries was entered into the calculated models because it was close to significance *p* < 0.10 (Table 3).

**Figure 2:**
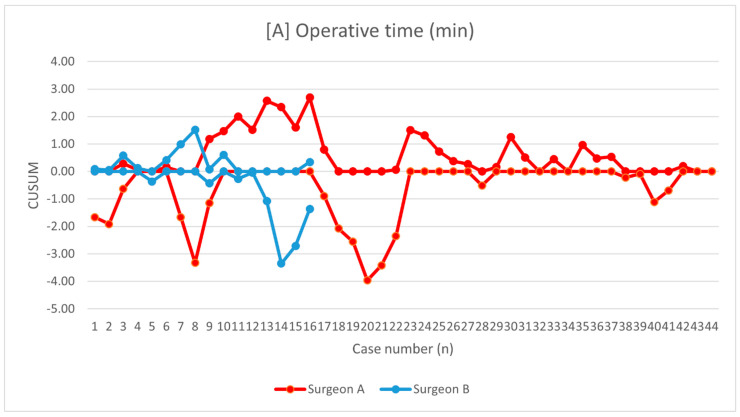
Chart showing the operative time presented as cumulative sum analysis separately for surgeons A and B.

**Figure 3:**
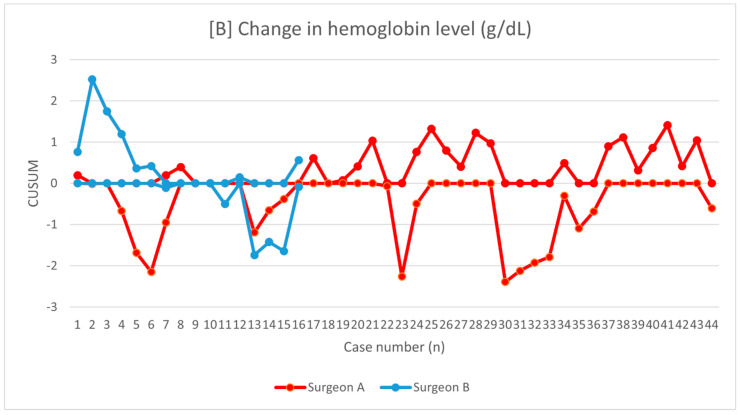
Chart showing the change in hemoglobin level presented as cumulative sum analysis separately for surgeons A and B.

**Figure 4:**
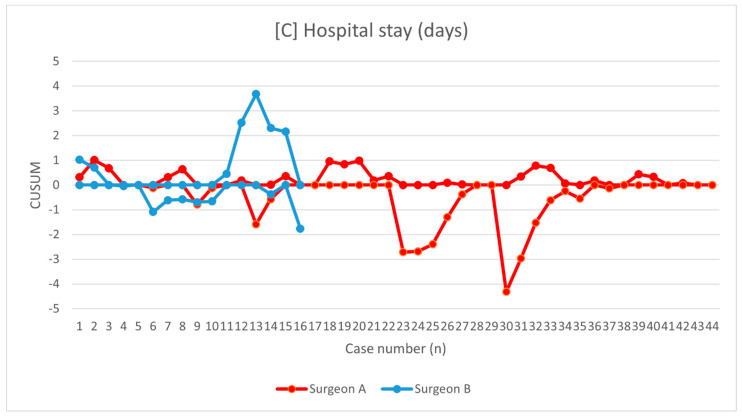
Chart showing the hospital stay presented as cumulative sum analysis separately for surgeons A and B.

The learning curves were demonstrated for operative time (Figure 2), change in hemoglobin level (Figure 3), and hospital stay (Figure 4). Mean operative time was 144.6 ± 21.2 min and 142.6 ± 27.5 min for surgeons A and B, respectively (*p* = 0.56). Using the KPSS test, we found that the operative time for surgeon A became stationary to his trend after 28 procedures (KPSS = 0.08; *p* > 0.10). The operative time varied up to the 28th case, and then became close to the average performance of surgeon A. The KPSS test was not performed for surgeon B because of a small group. The CUSUM operative time evaluation showed a turning point only for surgeon A after 38–40 procedures, which can be considered as the moment of steady proficiency level in this method. At the beginning of his training, deviations from the average operative time were significant; then with subsequent cases, they began to decrease, which allowed the learning process to be presented. The analysis of CUSUM surgeon A for hospital stay revealed a decreasing trend and plateaued after 41 cases. The curves for estimated blood loss for both surgeons showed fluctuations without a significant change below the zero line.

### 3.3. Perioperative Complications

In LSH(+), complications were reported in 7 cases of C–D grade I: subcutaneous emphysema of the left labia majora during laparoscopy, an injury around trocar insertion, whole body itching (which required intravenous antihistamine drugs), subconjunctival bleeding, bradycardia, one urinary tract infection after surgery, and the need for intravenous diuretics in 1 patient. In LSH(−), 2 cases of C–D grade I (atrial fibrillation, the need for intravenous diuretics) and 1 case of C–D grade IIIb complication were observed. The severe complications (≥III C–D grade) were low and appeared only in one patient in LSH(−) (bowel injury), which gives a severe complication rate of 1.7% for all LP performed. There was no correlation between the surgeon and the complication rate (*p* = 0.56). Higher level of complications was not observed in patients with history of supracervical or total hysterectomy (*p* = 0.37). Conversion to open pectopexy, return to the operating room or blood transfusion were not required. No C–D grade II or IV complications, and operative mortality (C–D grade V) were found.

## 4. Discussion

In our study, a proficiency based on CUSUM analysis was observed after 38–40 laparoscopic pectopexies, with a steady operative time achieved after 28 procedures. Studies about the learning curve for apical prolapse treatment mainly focus on LS, with operative time as the best indicator of mastering the procedure. The adequate performance for LS was reported after 60 cases, and operative time declined rapidly during the first 30 procedures, reaching a steady performance level after 90 patients [6]. In the case of a robot-assisted laparoscopic sacrocolpopexy (RASC), proficiency was achieved after 78 cases, and operative time decreased after 24–29 cases [7]. In another study about RASC, median operative time significantly reduced from 5.3 to 3.6 h during 7 years and plateaued after the first 60 cases [8].

Currently, LP is commonly performed in German initial centers, but there have been no reports about the number of the procedures needed to achieve proficiency yet [13]. Therefore, no comparisons on the learning curve with other centers could be performed. In a recently published multicenter study, mean operative time was 135 ± 46.08 min for combined pectopexy and 2–4 concurrent surgeries, and LP alone took 46.21 ± 18.47 min [13]. A similar time for the procedure was reported by the South Korean researchers with mean time 121.0 min [range 85–205] combined for LP and concurrent surgeries [18].

A CUSUM curve is a graphical representation of trends and outcomes of consecutively performed surgical procedures. According to the literature, complex procedures are more likely to follow gradual learning curves, and the improvement is achieved only after considerable experience. Steep learning curves imply that skills are acquired rapidly, usually because the procedure is simple [29]. The CUSUM curve showed that operative time stabilized after gaining experience by the surgeon, in our study, only for surgeon A, with a mean time of 144.6 ± 21.2 min for pectopexy combined with concurrent surgeries. The learning curve for estimated blood loss did not show a steady trend but remained within an acceptable range since the study’s commencement; we did not observe any intraoperative bleeding. It is worth emphasizing that in our study, we controlled the influence of independent variables such as BMI, concomitant procedures, previous POP surgeries, and LSH group membership on the dependent variables. The regression analysis allowed us to remove the “informational noise” and perform a precise analysis of the LP learning curve.

Apart from operative time, an even more important aspect of implementing a new procedure is the complication rate. Perioperative complications in our study were infrequent (*n* = 10; 16.7%), with one severe complication (1.7%) unrelated to pectopexy, which suggests that implementation of LP is safe. One severe complication did not allow us to determine a risk-adjusted CUSUM control chart of complications. To the best of our knowledge, the C–D classification has been used only in one study concerning LP so far [16]. Other authors reported varying numbers of complications after LP, from none [17,18] to 1 (3.6%) [12], 2 (5.6%) [15] and 6 (27.3%) [16]. In a multicenter trial, severe complications occurred in 5 (1%) cases, with the total count of 26 (5.2%). They reported bladder or ureter injuries, bleeding, and urinary tract infection [13]. Yet, the literature reports about serious complications during LP are rare, which is consistent with our results [12,13,15,16,17,18].

In our study LSH(−) group included both: patients after total hysterectomy and supracervical hysterectomy. It can be a limitation and implies difficulties in the pectopexy learning curve analysis. Although fixation of the mesh to the iliopectineal ligaments and the cervix or the vaginal cuff during LP, with or without concomitant LSH, is similar, two factors can prolong operative time: dissection of adhesions after previous removal of the uterus, and time-consuming procedures like morcellation of the uterine corpus. According to the literature, bag use (in/out) and morcellation lasts approximately 19.5 min, but with a wide range of 8 to 82 min [30]. In our study, we did not confirm a significant increase of operative time when morcellation was used. However, step by step timing of the procedure was not performed.

Apart from perioperative complications we did not analyze the subjective and anatomical success in this study. This is a limitation of the study aimed primarily to investigate the learning curve thoroughly. The detailed analysis of POP in particular compartments and lower urinary tract symptoms is an area for assessing the procedure’s long-term effects and will be a subject of our future research.

It is unclear whether our results considering the number of procedures to achieve surgical proficiency can be generalized to other hospitals. Both surgeons were very experienced, including in oncological procedures, before initializing LP in our center. In the multicenter study, the authors included surgeons who had performed at least 10 LP before entering the protocol [13]. Initial experience is of great importance if the learning curve is analyzed. The learning curve for surgeon B continues. However, we decided to publish our data at this point of the study as more centers report their initial results of LP repair. Our research can be a starting point for comparing the LP learning curves with other centers.

Analysis of the implementation of pectopexy for apical prolapse treatment at a tertiary center, the presence of monodisciplinary surgical team with experience in laparoscopy, and incorporation of perioperative complications according to the C–D classification constituted the strengths of the study. In the absence of studies describing the LP learning curve with CUSUM analysis and the KPSS test, our results provide compelling evidence.

In conclusion, stabilization of total operative time for LP based on the KPSS test was obtained after 28 cases. A moment of steady proficiency level based on CUSUM analysis was observed after 38–40 procedures. The pectopexy was associated with a low severe perioperative complication rate.

## Figures and Tables

**Table 1:** Characteristics of the patients operated by surgeon A and B and dichotomized into LSH(−) and LSH(+) groups.

	Surgeon A (*n* = 44)	Surgeon B (*n* = 16)	*p*	LSH(−) Pectopexy (*n* = 19)	LSH(+) Pectopexy with LSH (*n* = 41)	*p*
Age (years)	62.1 ± 8.4	63.7 ± 9.0	0.60 ^a^	62.3 ± 7.8	62.7 ± 8.9	0.61 ^a^
BMI (kg/m^2^)	27.6 ± 4.2	27.9 ± 4.2	0.86 ^a^	27.5 ± 4.2	27.7 ± 4.2	0.96 ^a^
Postmenopausal	39 (88.6%)	14 (87.5%)	0.90 ^b^	18 (94.7%)	35 (85.4%)	0.29 ^b^
Parity	2.4 ± 1.1	2.2 ± 1.2	0.44 ^a^	2.7 ± 1.5	2.1 ± 0.9	0.14 ^a^
Operative time (min)	144.6 ± 21.2	142.6 ± 27.5	0.56 ^a^	133 ± 22.8	149.1 ± 21.2	**0.01** ^**a**^
Pre-operative hemoglobin level (g/dL)	13.6 ± 1.0	13.2 ± 1.0	0.13 ^a^	13.5 ± 1.0	13.5 ± 0.9	0.84 ^a^
Post-operative hemoglobin level (g/dL)	12.1 ± 1.0	11.7 ± 0.8	0.14 ^a^	12 ± 1.1	12 ± 0.9	0.96 ^a^
Change in hemoglobin level (g/dL)	−1.5 ± 0.6	−1.5 ± 0.6	0.73 ^a^	−1.5 ± 0.6	−1.5 ± 0.5	0.74 ^a^
Hospital stay (days)	2.3 ± 0.6	2.8 ± 1.4	0.38 ^a^	2.4 ± 0.8	2.5 ± 1.0	0.81 ^a^
Pre-operative POP-Q stage			0.83 ^b^			**0.001** ^**b**^
II	1 (2.3%)	0		1 (5.3%)	0	
III	28 (63.6%)	12 (75.0%)		8 (42.1%)	36 (87.8%)	
IV	15 (34.1%)	4 (25.0%)		10 (52.6%)	5 (12.2%)	
Prior POP/UI surgery			0.24 ^b^			0.33 ^b^
Anterior colporrhaphy	0	1 (6.3%)		0	1 (2.4%)	
Posterior colporrhaphy	0	0		0	0	
Anterior and posterior colporrhaphy	7 (15.9%)	4 (25%)		1 (5.3%)	10 (24.4%)	
Kelly plication	1 (2.3%)	3 (18.8%)		0	4 (9.8%)	
Sacrospinous ligament suspension	4 (9.1%)	1 (6.3%)		2 (10.5%)	3 (7.3%)	
Transobturator tape	0	1 (6.3%)		0	1 (2.4%)	
Anterior vaginal repair with mesh	1 (2.3%)	0		1 (5.3%)	0	
Posterior vaginal repair with mesh	2 (4.5%)	0		1 (5.3%)	1 (2.4%)	
Total number in patients	9 (20.5%)	7 (43.8%)		2 (10.5%)	14 (34.1%)	
Prior uterine surgery			0.68 ^b^		X	X
TLH	0	0		0		
LSH	1 (2.3%)	0		1 (5.3%)		
TVH	4 (9.1%)	0		4 (21.1%)		
SH	6 (13.6%)	1 (6.3%)		7 (36.8%)		
TAH	6 (13.6%)	1 (6.3%)		7 (36.8%)		

Note: Values in bold represent statistically significant values. Data presented as mean ± standard deviation or *n* (%); ^a^—Mann-Whitney U test, ^b^—Pearson chi-square test BMI—body mass index, LSH—laparoscopic supracervical hysterectomy, LSH(−)—patients with history of supracervical or total hysterectomy, LSH(+)—patients with concomitant supracervical hysterectomy, POP-Q—Pelvic Organ Prolapse Quantification, SH—supracervical hysterectomy, TAH—total abdominal hysterectomy, TLH—total laparoscopic hysterectomy, TVH—total vaginal hysterectomy, UI—urinary incontinence.

**Table 2:** Concurrent procedures, reoperations, and perioperative complications according to the C–D classification in groups of patients.

	Surgeon A (*n* = 44)	Surgeon B (*n* = 16)	*p*	Pectopexy (*n* = 19)	Pectopexy with LSH (*n* = 41)	*p*
Concurrent procedures						
Bilateral salpingo-oophorectomy	28 (63.6%)	14 (87.5%)	0.07 ^a^	8 (42.1%)	28 (68.3%)	**0.001** ^**a**^
Unilateral/bilateral salpingectomy	4 (9.1%)	1 (6.3%)	0.21 ^a^	0	5 (12.2%)	0.16 ^a^
Unilateral/bilateral oophorectomy	3 (6.8%)	0	0.28 ^a^	3 (15.8%)	0	**0.009** ^**a**^
Perineal repair	0	2 (12.5%)	**0.017** ^**a**^	1 (5.3%)	1 (2.4%)	0.57 ^a^
Morcellation	6 (13.6%)	3 (18.8%)	0.62 ^a^	0	9 (22.0%)	<**0.03** ^**a**^
Complications according to the C–D classification						
None	38 (86.3%)	12 (75.0%)	0.56 ^a^	16 (84.2%)	34 (82.9%)	0.37 ^a^
I	5 (11.4%)	4 (25.0%)		2 (10.5%)	7 (17.1%)	
II	0	0		0	0	
III	1 (2.3%)	0		1 (5.3%)	0	
IV, V	0	0		0	0	

Note: Values in bold represent statistically significant values. Data presented as *n* (%), C–D—Clavien–Dindo classification, ^a^—Pearson chi-square test.

**Table 3:** Regression models for operative time, change in hemoglobin level and hospital stay.

Surgeon (*n*)	F	*p*	R^2^	ΔR^2^	Independent Variables	Dependent Variables	B	s.e.	β	t	*p*
A (44)	6.62	0.000	0.64	0.40	(Constant)	Operative time (min)	91.33	21.08		4.33	0.000
					**BMI**		**1.59**	**0.65**	**0.31**	**2.45**	**0.019**
					**Sum of the concurrent procedures**		**14.78**	**5.59**	**0.38**	**2.64**	**0.012**
					Previous POP surgeries		4.81	3.26	0.19	1.48	0.148
					Group [1-LSH(+), 2-LSH(−)]		−4.36	6.17	−0.10	−0.71	0.484
B (16)	1.96	0.171	0.65	0.42	(Constant)		217.27	53.66		4.05	0.002
					BMI		−0.37	1.59	−0.06	−0.23	0.822
					Sum of the concurrent procedures		−6.52	16.30	−0.10	−0.40	0.697
					Previous POP surgeries		−15.16	8.28	−0.45	−1.83	0.094
					**Group [1-LSH(+), 2-LSH(−)]**		**−42.03**	**19.02**	**−0.52**	**−2.21**	**0.049**
A (44)	0.47	0.761	0.05	−0.05	(Constant)	Change in hemoglobin level (g/dL)	−1.84	0.70		−2.64	0.012
					BMI		0.01	0.02	0.06	0.37	0.711
					Sum of the concurrent procedures		−0.01	0.18	−0.01	−0.08	0.938
					Previous POP surgeries		−0.12	0.11	−0.18	−1.08	0.285
					Group [1-LSH(+), 2-LSH(−)]		0.12	0.20	0.11	0.58	0.566
B (16)	3.16	0.059	0.53	0.37	(Constant)		−3.53	1.04		−3.41	0.006
					**BMI**		**0.07**	**0.03**	**0.49**	**2.29**	**0.043**
					Sum of the concurrent procedures		0.58	0.31	0.39	1.83	0.094
					Previous POP surgeries		0.02	0.16	0.03	0.14	0.895
					Group [1-LSH(+), 2-LSH(−)]		−0.55	0.37	−0.32	−1.49	0.163
A (44)	0.21	0.93	0.02	−0.08	(Constant)	Hospital stay (days)	2.65	0.78		3.41	0.002
					BMI		−0.01	0.02	−0.05	−0.28	0.784
					Sum of the concurrent procedures		−0.03	0.21	−0.03	−0.16	0.870
					Previous POP surgeries		−0.09	0.12	−0.12	−0.74	0.462
					Group [1-LSH(+), 2-LSH(−)]		−0.06	0.23	−0.05	−0.25	0.802
B (16)	1.29	0.331	0.32	0.07	(Constant)		4.11	2.93		1.40	0.189
					BMI		−0.14	0.09	−0.43	−1.65	0.127
					Sum of the concurrent procedures		1.43	0.89	0.41	1.60	0.138
					Previous POP surgeries		0.10	0.45	0.06	0.22	0.830
					Group [1-LSH(+), 2-LSH(−)]		0.79	1.04	0.19	0.76	0.463

Note: Values in bold represent statistically significant values. β = standardized regression, B—unstandardized regression coefficient, BMI—body mass index, LSH—laparoscopic supracervical hysterectomy, F—Fisher’s test statistic for analysis of variance (ANOVA), *p*—significance, R^2^—determination coefficient, ΔR^2^—adjusted determination coefficient, s.e.—standard error of unstandardized regression coefficient, t—Student’s t-test.

## Data Availability

The data presented in this study are available on request from the corresponding author. The data are not publicly available due to ongoing study.
